# AC5 protein encoded by squash leaf curl China virus is an RNA silencing suppressor and a virulence determinant

**DOI:** 10.3389/fmicb.2022.980147

**Published:** 2022-08-19

**Authors:** Huijie Wu, Mei Liu, Baoshan Kang, Liming Liu, Ni Hong, Bin Peng, Qinsheng Gu

**Affiliations:** ^1^Zhengzhou Fruit Research Institute, Chinese Academy of Agricultural Sciences, Zhengzhou, China; ^2^College of Plant Science and Technology, Huazhong Agricultural University/Key Lab of Plant Pathology of Hubei Province, Wuhan, China

**Keywords:** SLCCNV, AC5 protein, RNA silencing suppressor, virulence determinant, subcellular localization

## Abstract

Squash leaf curl China virus (SLCCNV) is a bipartite Begomovirus. The function of the protein AC5, which is encoded by SLCCNV, is unknown. Here, we confirmed that the 172-amino acids (aa) long AC5 protein of SLCCNV could suppress single-stranded RNA but not double-stranded RNA-induced post-transcriptional gene silencing (PTGS). Furthermore, we determined that the C-terminal domain (96–172 aa) of the AC5 protein was responsible for RNA silencing suppressor (RSS) activity *via* deletion mutant analysis. The AC5 protein can reverse GFP silencing and inhibit systemic silencing of GFP by interfering with the systemic spread of the GFP silencing signal. The SLCCNV AC5 protein was localized to both the nucleus and cytoplasm of *Nicotiana benthamiana* cells. Furthermore, deletion analysis showed that the putative nuclear localization signal (NLS, 102–155 aa) was crucial for the RNA silencing suppression activity of AC5. In addition, the AC5 protein elicited a hypersensitive response and enhanced potoao virus X (PVX) RNA accumulation in infected *N*. *benthamiana* plants. Using the infectious clones of the SLCCNV and SLCCNV-AC5 null mutants, mutational analysis confirmed that knockout of the *AC5* gene abolished SLCCNV-induced leaf curl symptoms, showing SLCCNV AC5 is also a virulence determinant.

## Introduction

The family *Geminiviridae* is the largest DNA virus family, which causes severe diseases and significant crop losses (Hanley-Bowdoin et al., 2013). It encompasses nine genera (Siddell et al., 2019). Begomoviruses are one of the largest and most successful plant viruses that could infect a wide range of crops, particularly in tropical and subtropical regions (Fontenellea et al., 2007). Begomoviruses possess either monopartite genome DNA A or bipartite genomes DNA A and B (Asigul et al., 2018). Squash leaf curl China virus (SLCCNV) belongs to the genus *Begomovirus* and is an important bipartite virus that causes severe economic losses in Cucurbitaceae crops such as wax gourd, squash, and pumpkin (Saritha et al., 2011; Riyaz et al., 2013; Maina et al., 2017). The disease incidence in ash gourds can reach 100% in the field (Riyaz et al., 2013). SLCCNV infection was first reported in melons in Hainan Province, China (Wu et al., 2020).

In recent years, several reports have shown that some begomoviruses contain an AC5 open reading frame (ORF), and their functions have been explored. For example, Watermelon chlorotic stunt virus (WmCSV) DNA-A AC5 from the complementary sense contains 255 amino acids (aa) but does not encode a functional protein (Kheyr-Pour et al., 2000). Tomato chlorotic mottle virus (ToCMoV-[MG-Bt]) AC5 gene encodes 250 amino acid residues and is not important for virus infection (Fontenellea et al., 2007). On the other hand, the Mungbean yellow mosaic India virus (MYMIV) AC5 protein contains 83 aa residues and has an RNA silencing suppressor (RSS) activity on post-transcriptional gene silencing (PTGS) or transcriptional gene silencing (TGS; Li et al., 2015). Ageratum leaf curl Sichuan virus (ALCScV) encodes a C5 protein with 115 aa residues and is a virulence factor that enhances the pathogenicity of PVX in *N. benthamiana* plants. Knockout of the AC5 gene significantly reduced disease symptoms and virus accumulation (Li et al., 2021).

SLCCNV encodes seven open reading frames (ORFs). Its DNA-A encodes *AV1*, *AC1*, *AC2*, *AC3*, and *AC5*, while the DNA-B encodes *BV1* and *BC1* proteins (Sawangjit, 2009; Fondong, 2013). SLCCNV AC5 contains 172 amino acids and is located on the complementary strand at nucleotide (nt) positions-825 to 309, downstream of the AC3 ORF and overlaps with the AV1 ORF on the viral sense strand. Prior to this study, the functions of SLCCNV AC5 were unknown. Here, we determined that the SLCCNV-encoded AC5 protein is a virulence determinant. In addition, the AC5 protein of SLCCNV was identified as an RSS of PTGS, and it did not inhibit inverted repeat RNA-induced PTGS (IR-PTGS). AC5 protein was localized in both the nucleus and cytoplasm of *N. benthamiana* cells. Deletion mutation analysis of the putative nuclear localization signal (NLS, aa 102–155) showed that AC5 was unable to localize to the nucleus if the NLS was deleted. Moreover, the NLS of AC5 was crucial for its local RNA silencing suppression activity. Furthermore, we confirmed that AC5 is a virulence determinant of SLCCNV infection in *N*. *benthamiana* plants. A comparison of the symptoms induced by wild-type SLCCNV and the SLCCNV-AC5 null mutant also confirmed that AC5 protein is a virulence determinant in melon plants.

## Materials and methods

### Materials and virus source

Wild-type *N. benthamiana* plants were used in this study. The SLCCNV infectious clone used was previously constructed in our laboratory (Wu et al., 2020). The melon cv. *Baimei* was maintained in an environmentally controlled, insect-free growth chamber at 28°C with a 16-h light and 8-h dark photoperiod. The GFP transgenic *N. benthamiana* 16c plants (Ruiz et al., 1998), p35S-GFP, p35S-dsFP (pCHF3-35S-dsFP), and p19 (Cymbidium ringspot virus p19 gene, 35S-p19; Lakatos et al., 2004; Xiong et al., 2009) were gifts from Professor Xueping Zhou of the Chinese Academy of Agricultural Sciences, Beijing, PR China.

### Construction of an SLCCNV-AC5 mutant with its translation initiation codon nullified

An AC5 gene with a nullified translation initiation codon (SLCCNV-AC5 null mutant DNA-A + DNA-B) was constructed using an SLCCNV DNA-A + DNA-B wild-type infectious clone (Wu et al., 2020). The AC5 gene did not overlap with AV1 but was located within the AV1 coding region. A T984C substitution was constructed into the translation initiation codon of AC5 (ATG-GTG) *via* site-directed mutagenesis to nullify AC5 translation initiation without affecting the aa sequence of its AV1 protein. The overlapping primers SLCCNV-AC5 null mutant-F/SLCCNV-AC5 null mutant-R were designed and used to amplify the full-length sequence of the AC5 null mutant gene. To inoculate melon plants, SLCCNV-AC5 null mutants were transfected into *Agrobacterium tumefaciens* GV3101 and then infiltrated into melon cotyledons along with SLCCNV DNA-B infectious clones. Wild-type SLCCNV infectious DNA-A and DNA-B clones were used as positive controls. The vector pCAMBIA1300 without the insert was used as the negative control. Symptoms were observed in inoculated melon plants at 14 day-post-inoculation (dpi).

Total DNA was extracted from melon plants infected with the SLCCNV-AC5 null mutant and SLCCNV wild-type infectious clones. The primers SLCCNV-A-F/SLCCNV-A-R and SLCCNV-B-F-478/SLCCNV-B-R-1284 (Supplementary Table 1) were used to obtain AV1 and BV1 probes to detect DNA-A and DNA-B, respectively. For Southern blot analysis of virus accumulation, 5 μg total DNA was separated on a 0.7% agarose gel and transferred to a Hybond-N+ membrane (Amersham, GE Healthcare Life Sciences) *via* capillary action, hybridized, and detected with the AV1 and BV1 sequence probes generated by a digoxigenin (DIG) High Prime DNA Labeling and Detection Starter Kit II (Roche, Switzerland), according to the manufacturer’s instructions. ImageJ software was used to analyze the accumulation levels of DNA-A and DNA-B in plants according to the methods described by Yang et al. (2020). The primers DNA-A-AV1-F1/ DNA-A-AV1-R1 DNA-B-BV1-F1/ DNA-B-BV1-R1 were used to detect viral accumulation in infected melon plants *via* quantitative PCR (qPCR), which were normalized to actin using the primers melonactin-F/melonactin-R, the actin genes.

### Plasmid construction of AC5 and its NLS deletion mutants

Primers were designed based on the genomic sequence of SLCCNV DNA A (HM566112.1). The primer sequences used in this study are listed in Supplementary Table 1. The pGreenII 62-SK vector was used as a backbone plasmid. The AC5 transient expression vector p35S-AC5 was also constructed. Several transient deletion expression mutants, p35S-AC5 (1–132 nt), p35S-AC5 (133–287 nt), and p35S-AC5 (288–519 nt) were also constructed.

Recombinant PCR was used to construct the pGR106-AC5 vector. The full-length sequence of AC5 (519 bp) was amplified *via* PCR using the primers pGR106-AC5-F and pGR106-AC5-R (Supplementary Table 1) and inserted into the Cla*I*/Sal*I* sites of the pGR106 vector to generate pGR106-AC5.

### *Agrobacterium*-infiltration and GFP expression

For PTGS experiments, classical two-component transient PTGS assays were performed as previously described (Li et al., 2015). Each culture, carrying one of these constructs: p35S-AC5, p35S-AC5 (1–132 nt), p35S-AC5 (133–287 nt), or p35S-AC5 (288–519 nt), with p35S-GFP, was co-infiltrated into the leaves of GFP-transgenic *N. benthamiana* 16c plants. p19 and an empty vector were used as positive and negative controls, respectively. Each inoculum was adjusted to an optical density at 600 nm (OD_600_) = 1.0 before infiltration. The ratio of p35S-GFP to the vector was 1:1. Infiltrated leaves were collected under UV light at 4 dpi. To further analyze whether AC5 interferes with the systemic gene silencing signal, GFP-transgenic *N*. *benthamiana* 16c plants co-infiltrated with the above vectors, p19, and an empty vector as a control, were tested.

For IR-PTGS experiments, following a method previously described (Li et al., 2015), *A*. *tumefaciens* cultures harboring p35S-GFP, p35S-dsFP, and p35S-AC5 vectors were mixed in equal proportions (1:1:1) and infiltrated into *N*. *benthamiana* wild-type leaves. The p19 and empty vector pGreenII 62-SK (Sangon Biotech Co., Ltd., Shanghai, China) were used as the positive and negative controls, respectively. Infiltrated leaves were observed and collected at 5 dpi under UV light.

For each experiment, all plants in the seven-leaf stage were infiltrated. The plants were grown in a growth chamber at 28°C with a 16-h light and 8-h dark photoperiod. Ten plants were infiltrated, and each treatment was repeated three times. GFP fluorescence was observed using a 100-W long-wave UV lamp (Black Ray Model B 100A, UV products). The leaves or whole plants were photographed.

### RNA analyses

Total RNA was extracted using an RNAprep pure plant kit (DP432) from Tiangen Biotech Co., Ltd. (Beijing, China), according to the manufacturer’s instructions. The primer pairs GFP-probe -F/T7-GFP-probe -R, AC5-probe -F/T7-AC5-probe –R, and PVX-cp-F/ PVX-cp-T7-R were used to prepare DIG-labeled DNA probes of the *gfp*, *AC5*, and PVX *cp* genes, respectively, using a DIG High Prime DNA Labeling and Detection Starter Kit (Roche, Switzerland). For northern blot analysis of GFP mRNA, AC5, and PVX cp expression levels, 5 μg of total RNA was separated on a 1.5% agarose-formaldehyde gel, transferred to a Hybond-N+ membrane (Amersham, GE Healthcare Life Sciences) *via* capillary action, hybridized, and detected using a DIG Nucleic Acid Detection Kit (Roche, Switzerland), according to the manufacturer’s instructions.

For real-time qPCR, total RNA was reverse-transcribed to complementary DNA (cDNA) using a HiScript II Q RT SuperMix for qPCR (+gDNA wiper; Vazyme, China). qPCR was conducted using a FastStart Universal SYBR Green Master (ROX; Roche, Switzerland) on a CFX 96 Real-Time System (Bio-Rad). The primers PVX cp-F/PVX cp-R were used to detect PVX mRNA expression. The primers Nb-actin-F/Nb-actin-R were used to amplify the actin gene, which was used as an internal control for the assays (Li et al., 2021). All gene expression data were analyzed in three biological and technical replicates each. Relative gene expression levels were calculated using the 2^-ΔΔCt^ method for analysis.

Next, siRNA blotting, hybridization, and detection of siRNA were performed as described previously, with minor modifications (Asigul et al., 2018). Briefly, 20 μg total RNA was separated on a 15% polyacrylamide/7 M urea gel and transferred onto a Hybond-N+ membrane (GE Healthcare). Membranes were hybridized with a DIG-labeled GFP siRNA probe (Sangon Biotech Co., Ltd.). A U6 probe (5′-TCC CAA TTC TTG AAT TAG-3′) labeled with DIG biotin at the 5′-end was synthesized as a control. The six primers G2, G3, F1, F2, F3, and P1 probes of GFP with a DIG-labeled biotin at the 5′-end were used to detect GFP siRNA (Supplementary Table 1).

### Detection of H_2_O_2_ in plants

H_2_O_2_ was detected in the leaves using the 3,3-diaminobenzidine (DAB)-HCl (Sigma) uptake method (Sharma and Ikegami, 2010). Briefly, leaves were excised from the base of the stems with a 1 mg/ml solution of DAB dissolved in Tris buffer (pH 3.8). After 4 h incubation in the light at 28°C, the leaves were immersed in 96% ethanol and boiled for 10 min. This treatment decolorized the leaves, except for the deep-brown polymerization product produced by the reaction of DAB with H_2_O_2_. After cooling, the leaves were preserved at 25°C in 70% ethanol and photographed.

### Western blot analysis

Total protein was extracted from the leaves of *N*. *benthamiana* plants as previously described (Ocampo et al., 2015) and western blotting was performed as previously described (Lu et al., 2018). The total protein was homogenized in a 2 × protein gel loading buffer (Amresco, Cat # E270-1 ml) at a 1:1 ratio(v/v). After boiling for 10 min and incubating on ice for 3 min, the protein samples were separated *via* 10% SDS-PAGE, and transferred to membrane by trans-blot system (Bio-Rad), then probing the membranes with anti-GFP monoclonal antibodies (Sigma-Aldrich, Cat #SAB2702211-100UL). Immunoreactive bands were visualized using an ECL western blotting detection kit (GE Healthcare) with a horseradish peroxidase (HRP) conjugated secondary antibody, following the manufacturer’s instructions. Images were acquired using a Tanon 5,200 Multi-Imaging System (Tanon, Shanghai, China).

### Subcellular localization of the AC5 protein

For subcellular localization assays of the AC5 protein, the GFP sequence was added to the N-terminus of AC5 *via* PCR and named p35s-AC5-GFP. A pair of primers (AC5^Δ102–115 aa^-F/AC5^Δ102–115 aa^-R; Supplementary Table 1) was used to amplify p35S-AC5^Δ102–115 aa^-GFP. An RFP-labeled nuclear marker, histone 2 B (H2B), was constructed as p35S-H2B-RFP and used as a positive control, while p35S-GFP was used as a negative control. Fluorescence analysis was performed using a Leica TC SSP5 confocal laser scanning microscope (Leica Microsystems, Heidelberg, Germany). GFP was excited at 488 nm and the emitted light was captured between 515 and 527 nm. The inoculated leaves were analyzed three times in each experiment.

## Results

### Phylogenetic analysis and conserved domains of the SLCCNV AC5 protein

**Figure 1:**
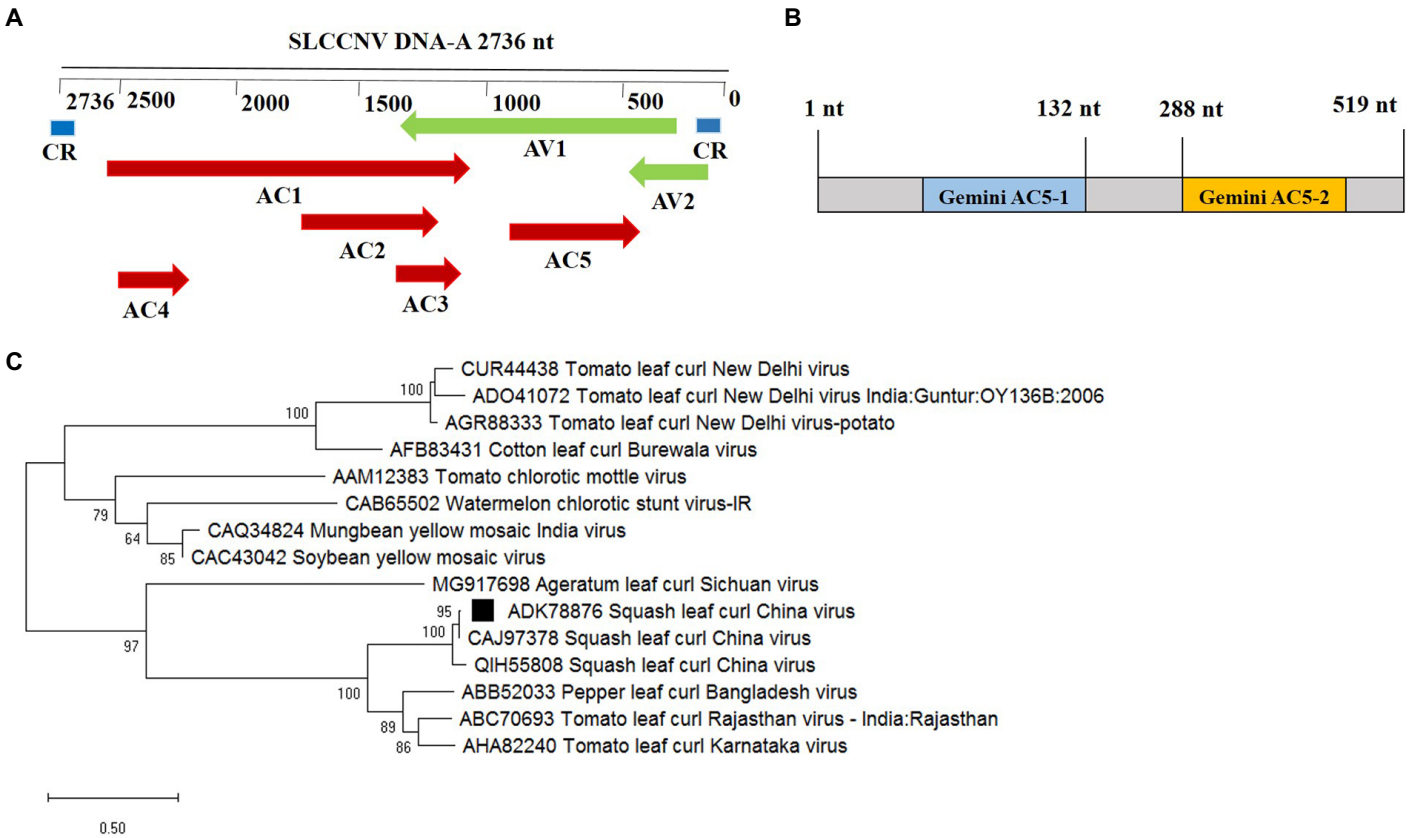
Phylogenetic analysis and domain structure of the squash leaf curl China virus (SLCCNV) AC5 protein. **(A)** The genomic organization of SLCCNV DNA-A. The common region (CR) and all the putative open reading frames (ORFs) located in the viral and complementary strands are indicated. **(B)** The conserved domain structures Gemini AC5-1 and Gemini AC5-2 were found in SLCCNV AC5 proteins, as determined using the conserved domain database (CDD). Note that the schematic domains of the AC5 protein may not be proportional to the aa scale bar shown at the top because of the varying lengths of the AC5 proteins. **(C)** Phylogenetic relationships of the AC5 amino acid (aa) sequences of representative begomoviruses. The AC5 aa sequences from begomoviruses were aligned using the Neighbor-Joining method in the MEGA6 program with 1,000 replications. Hides values were lower than 50%.

The SLCCNV AC5 ORF nucleotides (nt) 309–825 encode a 172-aa long protein in the complementary strand, which completely overlaps with the virion-sense CP ORF (nt 279–1,047; Figure 1A). Among the sequenced isolates of SLCCNV, the AC5 ORF was highly conserved in both position and aa sequence and contained two conserved domains: Gemini AC5-1 and Gemini AC5-2 (Figure 1B).^1^ The AC5 ORF is conserved and has a similar domain structure in many begomoviruses, suggesting that AC5 plays a functional role in plants during SLCCNV infection. To determine the evolutionary relationships of the AC5 protein from different begomoviruses, the phylogenetic relationship of the complete AC5 aa sequences of *Begomovirus* species from GenBank was analyzed. Phylogenetic trees were constructed using the neighbor-joining algorithm with 1,000 bootstrap replicates available in MEGA6 (version 6.0; Tamura et al., 2013). Two major branches were observed. SLCCNV appeared in an independent branch containing bipartite Begomoviruses, including several SLCCNV isolates. AC5 clustered with cucurbit crop isolates from China and Vietnam (Figure 1C).

### AC5 suppresses single-stranded RNA-triggered RNA silencing and its C-terminal domain is essential for its RSS activity

**Figure 2:**
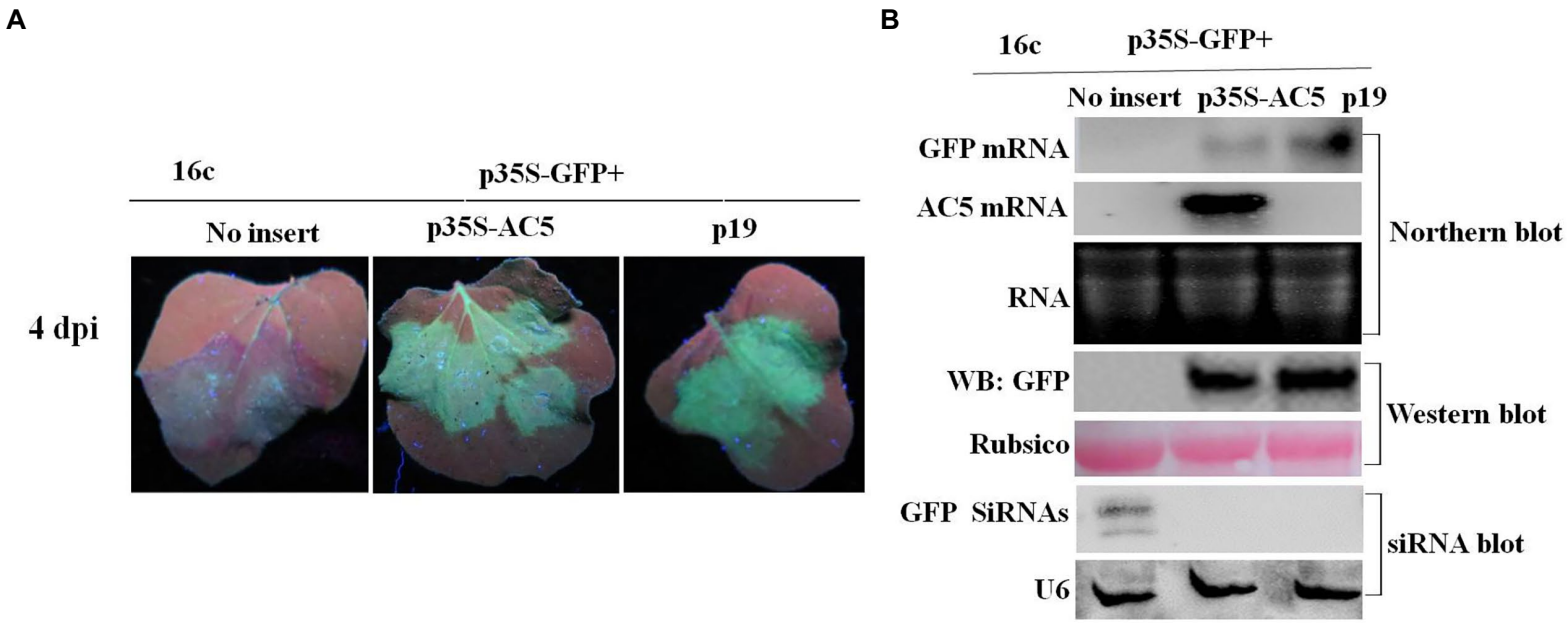
GFP silencing in GFP-transgenic *N*. *benthamiana* 16c plants. **(A)** Representative leaf patches or plants were co-infiltrated with *A*. *tumefaciens* cultures harboring the p35S-GFP, p35S-AC5, and expression vectors pGreenII 62-SK or the p19 vector. Images were photographed under UV light at 4 dpi. **(B)** Northern blot of GFP and AC5 mRNA accumulation, western blot of GFP protein accumulation and siRNA blot of GFP siRNA accumulation in *Agrobacterium*-infiltrated leaf patches as indicated in **(A)**. SYBR Safe DNA Gel staining of rRNA and Ponceau S staining of the subunit of Rubisco served as loading controls. U6 was the loading control in the siRNA blot.

The intensity of green fluorescence decreased in leaf patches infiltrated with the vector but increased in patches expressing the AC5 and p19 proteins. The leaves co-infiltrated with p19 and p35S-GFP showed stronger green fluorescence than the leaves infiltrated with the empty vector alone. The green fluorescence intensities of leaf patches infiltrated with p35S-AC5 + p35S-GFP were weaker than those infiltrated with p19 + p35S-GFP but significantly stronger than those of the empty vector control (Figure 2A). These results suggest that SLCCNV AC5 is an RSS of PTGS.

To confirm the phenotype observed, the levels of GFP mRNA, siRNAs, and proteins were analyzed. Northern blotting revealed that local silencing suppression of AC5 and p19 was confirmed by the accumulation of GFP mRNA and the loss of GFP-specific siRNAs in infiltrated leaves. As shown in Figure 2B, AC5 and p19 appeared to have the same GFP fluorescence intensity under UV light. Moreover, the expression level of AC5 was lower than that of p19, as determined *via* northern blotting. In addition, GFP siRNA accumulation was not detected in p35S-AC5 + p35S-GFP- and p19 + p35S-GFP-treated leaves but clearly detected in vector+p35S-GFP-treated leaves. Western blot analysis showed that the GFP expression was detected in p35S-AC5 + p35S-GFP- and p19 + p35S-GFP-treated leaves, with that in p19 + p35S-GFP-treated leaves being higher than those treated with p35S-AC5 + p35S-GFP. At the same time, we tested the AC5 mRNA levels in the leaves. AC5 mRNA expression was not detected in leaves infiltrated with the vector+p35S-GFP or p19 + p35S-GFP, but was very high in p35S-AC5 + p35S-GFP-treated leaves. Collectively, these data demonstrated that SLCCNV AC5 is an RNA-silencing suppressor of PTGS, with a weaker activity than the p19 protein.

**Figure 3:**
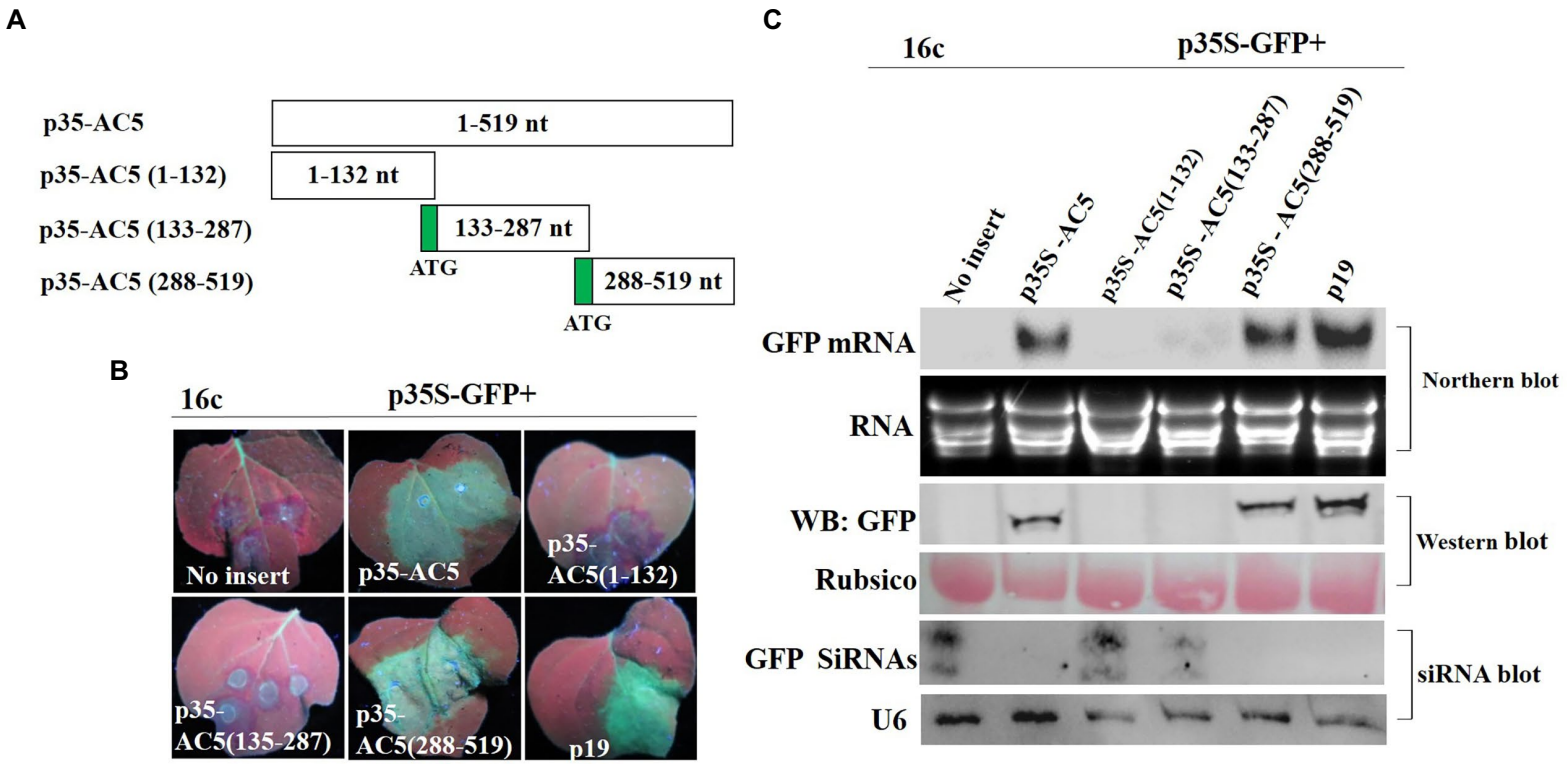
Identification of the AC5 region with RNA silencing suppressor activity. **(A)** Diagrams of the AC5 mutants. **(B)** Leaf patches were co-infiltrated with *A*. *tumefaciens* cultures harboring the p35S-GFP and empty pGreenII 62-SK vector, p35S-GFP + p35S-AC5, p35S-GFP + p35S-AC5(1–132), p35S-GFP + p35S-AC5(135–187), p35S-GFP + p35S-AC5(188–519), p35S-GFP + P19, p35S-AC5 and its mutants were photographed under UV light at 4 dpi. **(C)** Northern blot of GFP accumulation, GFP protein accumulation and siRNA blot of GFP siRNA accumulation in the *Agrobacterium*-infiltrated leaf patches as indicated in **(B)**. SYBR Safe DNA Gel staining of rRNA and Ponceau S staining of the subunit of Rubisco served as loading controls. U6 was the loading control in the siRNA blot.

To confirm which region of AC5 was responsible for its RSS activity, p35S-AC5 (1–132 nt), p35S-AC5 (132–287 nt), and p35S-AC5 (288–519 nt) were generated using the 35S promoter expression vector (Figure 3A). At 4 dpi, there was no GFP fluorescence in patches co-infiltrated with vector + p35S-GFP, p35S-AC5 (1–132 nt) + p35S-GFP, and p35S-AC5 (132–287 nt) + p35S-GFP. However, patches co-infiltrated with p35S-AC5 (288–519 nt) + p35S-GFP and p35S-AC5 + p35S-GFP showed similar fluorescence intensity but were weaker than patches treated with p19 + p35S-GFP (Figure 3B).

In Figure 3C, the GFP fluorescence was detected in *Agrobacterium*-infiltrated leaves. Northern blotting and western blotting showed the accumulation of GFP mRNA and protein. The p35S-AC5 + p35S-GFP and p35S-AC5 (288–519 nt) + p35S-GFP-infiltrated leaves showed significantly lower GFP mRNA and protein accumulation compared to p19-infiltrated leaves. Analysis of GFP small-RNA accumulation revealed that RNA silencing of GFP resulted in siRNA production. The results showed that the active site of the local silencing suppressor of AC5 resided at 288–519 nt, including the complete C-terminal conserved functional domain Gemini AC5-2 (Li et al., 2015).

### AC5 cannot suppress double-stranded RNA-triggered RNA silencing

**Figure 4:**
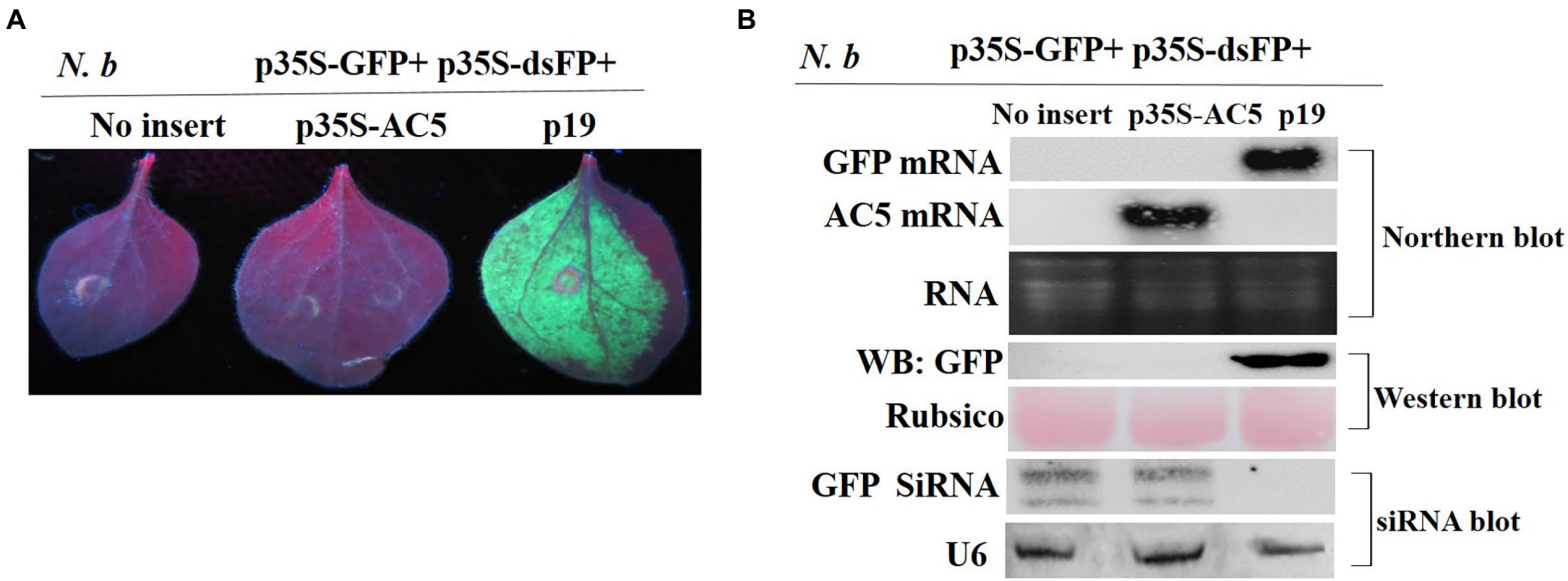
The SLCCNV AC5 could not inhibit GFP silencing of double-stranded dsRNA. **(A)** Representative 16c leaves were co-infiltrated with *A*. *tumefaciens* cultures harboring the p35S-GFP, double-stranded GFP (p35S-dsFP) and vector pGreenII 62-SK, p35S-AC5-expressing vectors or p19. Images were photographed under UV light at 4 dpi. **(B)** Northern blot of GFP and AC5 mRNA accumulation, western blot of GFP protein accumulation, and siRNA blot of GFP siRNA accumulation in *Agrobacterium*-infiltrated leaf patches. Ethidium bromide staining of rRNA and Ponceau S staining of the subunit of Rubisco served as loading controls. U6 was the loading control in the siRNA blot.

To determine whether the AC5 protein can block dsRNA-triggered RNA silencing, wild-type *N. benthamiana* leaves were infiltrated with *A*. *tumefaciens* containing p35S-GFP + p35S-dsFP, along with an empty vector, and constructs containing p35S-AC5 and p19. As shown in Figure 4A, leaves infiltrated with the vector control or p35S-AC5 and the silencing inducer (p35S-GFP + p35S-dsFP) lost GFP fluorescence at 4 dpi, indicating that dsRNA-induced RNA silencing was not suppressed by AC5. In contrast, enhanced GFP fluorescence was sustained in patches infiltrated with the positive control p19.

As shown in Figure 4B, no GFP mRNA transcript or protein was detected in leaf patches co-infiltrated with the vector control pGreenII 62-SK + p35S-GFP + p35S-dsFP or p35S-AC5+ p35S-GFP+ p35S-dsFP. GFP mRNA was degraded by the expression of dsGFP. High AC5 mRNA levels were detected in patches infiltrated with p35S-AC5 + p35S-GFP+ p35S-dsFP. In contrast, GFP mRNA and protein levels were high in the leaves infiltrated with p35S-GFP + p35S-dsFP+p19. Taken together, these results demonstrated that AC5 did not trigger dsRNA-induced GFP gene silencing in *N*. *benthamiana* plants.

### Interference of the AC5-induced systemic spread of RNA silencing

**Figure 5:**
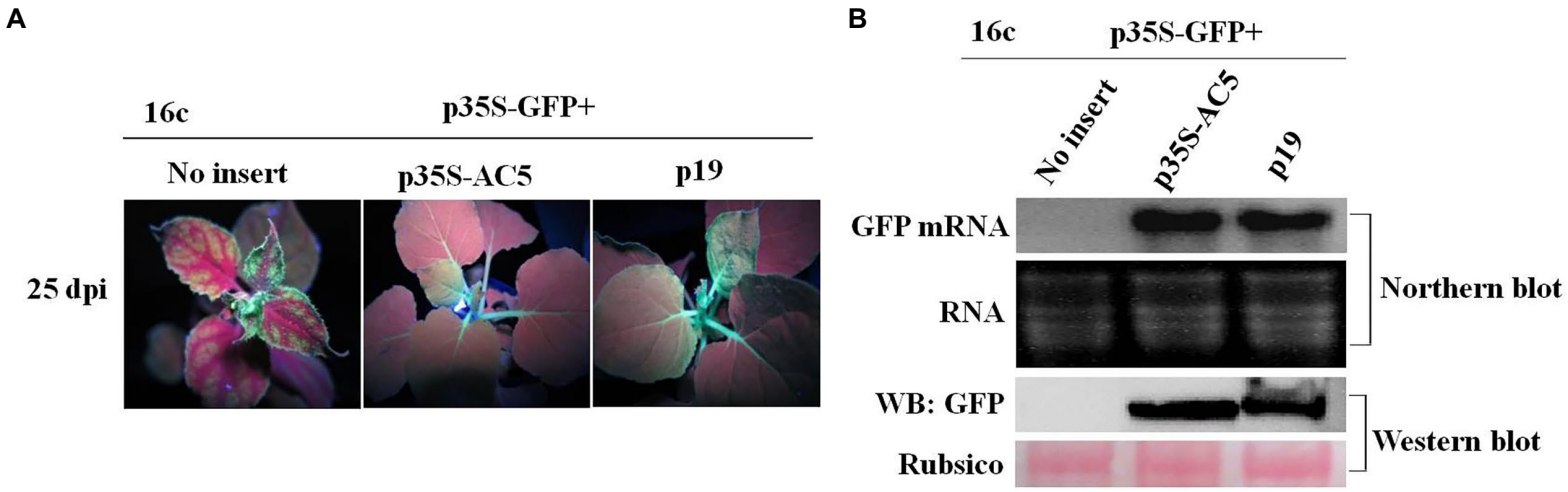
The effect of SLCCNV AC5 on suppressing systemic RNA silencing. **(A)** GFP-transgenic 16c plants were used to determine systemic silencing at 25 dpi. The GFP-transgenic 16c plants co-infiltrated at the seven-leaf stage with the mixture of carrying p35S-GFP, vector, p35S-AC5 expressing vectors or p19 were observed. **(B)** Northern blot analysis of GFP mRNA and western blot analyses of GFP, RNA, and proteins isolated from the systemic leaves at 25 dpi. SYBR Safe DNA Gel staining of rRNA and Ponceau S staining of the subunit of Rubisco served as loading controls.

We then tested whether SLCCNV AC5 systemically suppresses single-stranded GFP. *Agrobacterium*-infiltrated plants suppressed systemic silencing in upper young leaves at 15, 25, and 35 dpi (Table 1). At 25 dpi, symptoms induced by silencing showed that the major and minor veins of the uppermost young leaves turned red under UV light when treated with p35S-GFP + vector negative control. The uppermost young leaves were green under UV light after infiltration with p35S-GFP + p35S-AC5 and p35S-GFP + p19 (Figure 5A). GFP fluorescence was lost in 65.9% of leaves infiltrated with p35S-GFP+ empty vector. In contrast, GFP fluorescence persisted in most leaves from GFP-transgenic 16c plants infiltrated with p35S-GFP + p19 or p35S-GFP + AC5, with only 4.5 and 8.0% of the plants displaying systemic silencing, respectively. At 35 dpi, systemic GFP silencing was observed in approximately 92.0% of p35S-GFP+ vector-co-infiltrated plants, 18.4% in p35S-GFP + p35S-AC5 co-infiltrated plants, and 10.1% in p35S-GFP + p19 co-infiltrated plants (Table 1).

**Table 1:** The SLCCNV AC5 protein interfered spread of GFP RNA systemic silencing signal in the GFP transgenic 16c plants.

Treatments	Expression vectors	15 dpi	25 dpi	35 dpi
I	p35S-GFP+ empty vector	5/30 ^a^ 6/30 5/30	21/30 18/29 19/29	26/30 28/29 27/29
		17.8%	65.9%	92.0%
II	p35S-GFP+ P19	0/30 0/30 0/29	1/30 2/30 1/29	4/30 3/30 2/29
		0	4.5%	10.1%
III	p35S-GFP+ AC5	0/30 1/29 0/30	2/30 2/29 3/29	3/29 5/29 8/29
		1.1%	8.0%	18.4%

a Number of plants showing systemic GFP silencing/total number of plants tested. Each treatment was performed three times in the GFP transgenic 16c line plants. Values represent the mean number of systemic GFP silencing plants per 30 plants.

Northern blotting of GFP mRNA and western blotting of GFP protein in uppermost young leaves showed high accumulation in plants infiltrated with p35S-GFP + p35S-AC5 and p35S-GFP + p19. Similarly, AC5 mRNA accumulation was detected in plants infiltrated with p35S-GFP + p35S-AC5 (Figure 5B), but not in p35S-GFP + p35S-AC5 and p35S-GFP + p19. These results showed that AC5 interfered with the systemic spread of the gene-silencing signal.

### Subcellular localization of AC5 and the roles of its NLS

**Figure 6:**
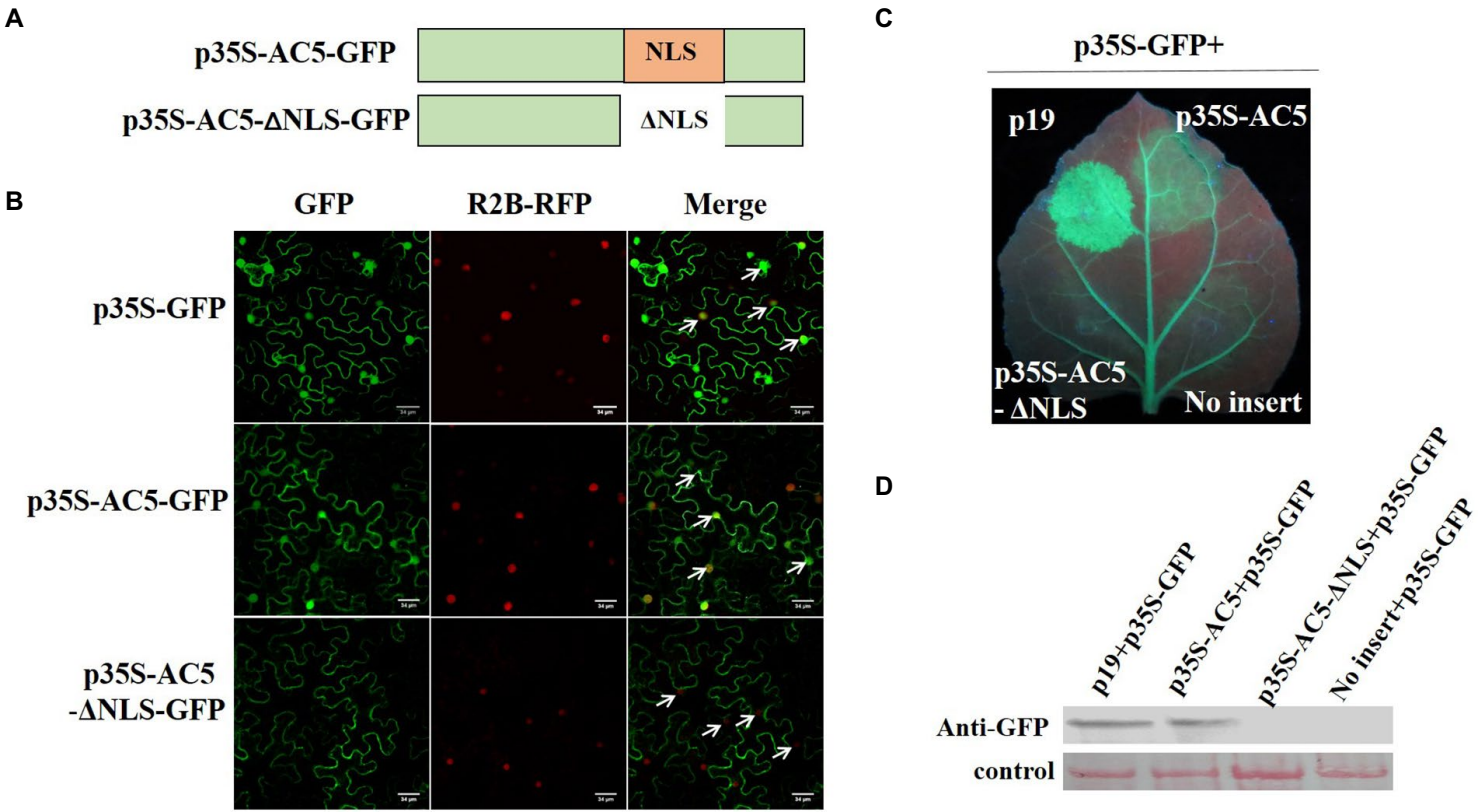
Analysis of the subcellular localization of SLCCNV AC5, the AC5 nuclear localization signal (NLS), and PTGS. **(A)** Schematic representation of SLCCNV AC5 and a deletion mutant of its NLS. **(B)** Cellular and subcellular localization of SLCCNV AC5 and its derivatives in cells. H_2_B-RFP represents RFP fused at the C-terminus of the nuclear marker histone 2B. The white arrows indicate the nuclei. Scale bar = 34 μm. **(C)** Suppression of GFP silencing in GFP-transgenic 16c plants. Leaf patches were co-infiltrated with *A*. *tumefaciens* cultures harboring p35S-GFP + p19, p35S-GFP + p35S-AC5, p35S-GFP+ p35S-AC5-Δ102-115 aa, and p35S-GFP+ empty vector. The leaves were photographed under UV light at 4 dpi. **(D)** Western blot analyses of dsRNA-induced GFP gene silencing in infiltrated leaf samples.

To analyze which region of AC5 contained the nuclear localization signal (NLS), its sequence was analyzed using the online bioinformatics tool NLS Mapper^2^ with a test score > 2. The predicted results showed that the regions covering aa 102–115 (IVPRFKRLNFTRAF) might be NLSs of the AC5 protein (Figure 6A). For subcellular localization, GFP distribution in leaves infiltrated with p35S-AC5-GFP was the same as that of free GFP. To confirm the nuclear localization signal (102–115 aa) of AC5, a nuclear localization signal deletion mutant, p35S-AC5-^Δ102-115 aa^-GFP, was shown to localize in the cytoplasm but not in the nucleus (Figure 6B). The results indicated that the AC5 protein of wild-type SLCCNV was localized in both the cytoplasm and the nucleus of *N*. *benthamiana* plants.

To investigate whether the NLS of SLCCNV AC5 can also suppress RNA silencing, we co-infiltrated p19 + p35S-GFP, p35S-AC5 + p35S-GFP, p35S-AC5-Δ102-115aa + p35S-GFP, and vector +p35S-GFP in the same GFP-transgenic 16c plant leaves. We observed that SLCCNV AC5 and p19 suppressed GFP silencing. However, the mutant p35S-AC5-Δ102-115 with its NLS (aa 102–115) deleted was unable to suppress GFP silencing (Figure 6C). The GFP expression levels of p19, p35S-AC5, p35S-AC5-Δ102-115 aa, and the empty vector pGreenII 62-SK in the GFP-transgenic 16c plants were determined *via* western blot analysis. The accumulation of GFP correlated with the intensity of GFP fluorescence (Figure 6D). Therefore, the results showed that the SLCCNV AC5-mediated RNA silencing suppression disappeared with the deletion of the NLS. Moreover, the NLS was located at 102–115 aa in the C-terminal Gemini AC5-2 domain (Li et al., 2015), further confirming that the RNA silencing suppression activity of the SLCCNV AC5 protein was located at its C-terminus.

### The AC5 protein elicits a hypersensitive response in *Nicotiana* *benthamiana* plants

**Figure 7:**
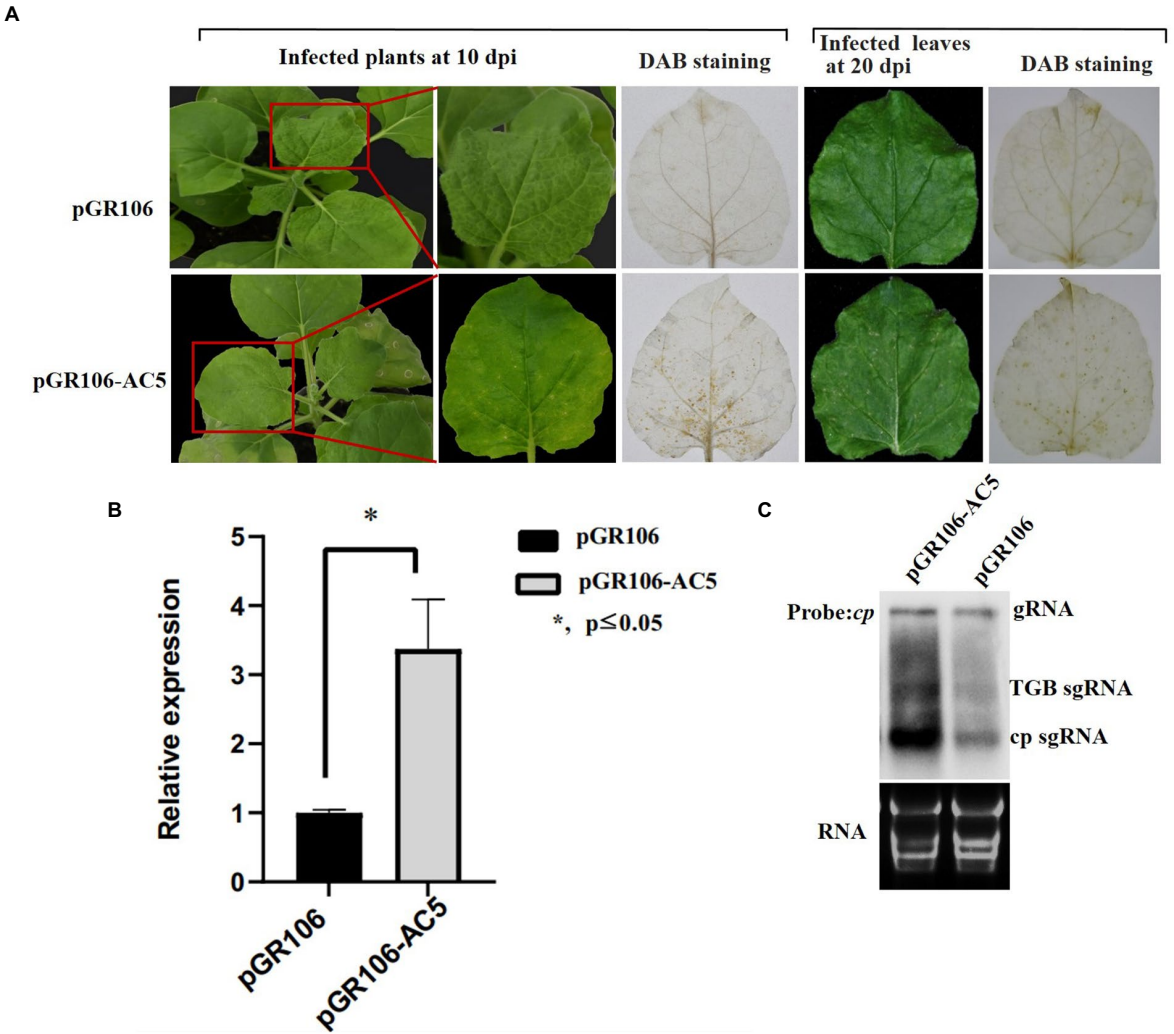
Symptoms exhibited by plants following inoculation with pGR106 or pGR106-AC5. **(A)** *N*. *benthamiana* plants inoculated with pGR106 or pGR106-AC5 were photographed at 10 day-post *Agrobacterium*-infiltration (dpai). Upper infected leaves were photographed directly at 10 dpi and 20 dpi or photographed after 3,3′- diaminobenzidine (DAB) staining. **(B)** Leaf tissues were harvested from pGR106- or pGR106-AC5-systematically infected *N*. *benthamiana* plants at 10 dpai. The mRNA accumulation of potato virus X (PVX) was analyzed using real-time qPCR. ^*^p ≤ 0.05, Student’s *t*-test. **(C)** Northern blot was used to detect mRNA accumulation, with the PVX *cp* gene as probe. The positions of genomic RNA (gRNA), TGB subgenomic RNA (TGB sgRNA), CP subgenomic RNA (CP sgRNA) were indicated.

To determine whether the AC5 protein is a virulence determinant in *N*. *benthamiana* plants, plants were tested with inoculation of pGR106-AC5 transcripts. Inoculated plants showed severe systemic chlorotic and necrotic spots in the newly emerged upper leaves. *N*. *benthamiana* plants inoculated with the pGR106 vector without AC5 protein exhibited mild systemic mosaic symptoms in the upper leaves at 8 dpi (Figure 7A). To ascertain whether the cell death or necrotic spots on *N*. *benthamiana* plants inoculated with pGR106-AC5 were caused by the accumulation of H_2_O_2_, plants infiltrated with either pGR106 or pGR106-AC5 vector were analyzed using the 3,3-diaminobenzidine (DAB) method (Sharma and Ikegami, 2010). The results showed that high H_2_O_2_ levels accumulated in the upper systemic leaves, showing chlorotic and necrotic spots in *N*. *benthamiana* plants infected with pGR106-AC5. At the same time, H_2_O_2_ accumulation was not detected in leaves infiltrated with pGR106 at 10 and 20 dpi (Figure 7A). These results demonstrated that AC5 is a virulence determinant and elicitor of cell death in *N*. *benthamiana* plants. Taken together, our results revealed that the AC5 protein caused more severe symptoms and is a candidate host defense response target. To further confirm the effect of AC5 on viral accumulation, northern blotting and real-time qPCR (RT-qPCR) were performed to analyze viral accumulation in the upper leaves of the pGR106-AC5- and pGR106-inoculated plants. The results showed that the expression level of PVX *cp* was higher in pGR106-AC5-inoculated plants than in pGR106-inoculated plants. Moreover, there was a significant difference in the expression levels between them, as determined by Student’s *t*-test (^*^p ≤ 0.05; Figure 7B). Furthermore, we found that the mRNA accumulation of pGR106-AC5 was higher than that of pGR106 using northern blotting (Figure 7C). Therefore, we identified AC5 as a virulence determinant of SLCCNV in *N*. *benthamiana* plants.

### The AC5 protein is a virulence determinant in melon plants

**Figure 8:**
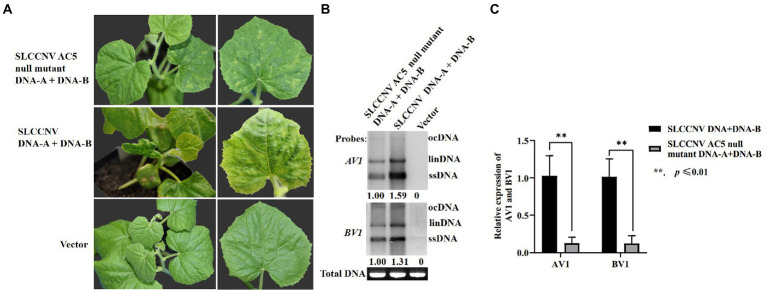
Symptoms of melon plants infected with SLCCNV and SLCCNV-AC5 null mutants. **(A)** Chlorotic mosaic symptoms of melon plants infected with SLCCNV-AC5 null mutant and DNA-B infectious clones (SLCCNV AC5 null mutant DNA-A + DNA-B). Curling and chlorotic mosaic symptoms were observed in melon plants infected with SLCCNV DNA-A and DNA-B wild-type infectious clones (SLCCNV DNA + DNA-B). No symptoms were observed in plants inoculated with an empty vector (negative control). **(B)** Southern blot analysis of viral accumulation. Leaves inoculated with SLCCNV AC5 null mutant DNA-A + DNA-B, and SLCCNV wild-type were detected at 14 dpi using DNA-A *AV1* and DNA-B *BV1* genes as probes. The empty vector served as the negative control. Viral open circular (ocDNA), linear (linDNA), and single-stranded DNAs (ssDNA) are indicated. ImageJ software was used to analyze the relative ssDNA expression of DNA-A *AV1* and DNA-B *BV1* in infected melon plants. **(C)** Viral DNA accumulation in the SLCCNV-AC5 null mutant DNA-A + DNA-B infected plants and the SLCCNV wild-type DNA-A + DNA-B infected melon plants was detected through quantitative PCR (qPCR). ^**^p ≤ 0.01, Student’s *t*-test.

To further verify whether AC5 is a virulence determinant of SLCCNV, an infectious clone of the SLCCNV-AC5 null mutant was constructed. Melon plants inoculated with the SLCCNV-AC5 null mutant and SLCCNV-DNA-B showed moderate chlorotic mosaic symptoms, loss of leaf curling, and dwarfing symptoms at 14 dpi. However, severe leaf curling, dwarfing, and chlorotic mosaic symptoms were observed in melon plants inoculated with SLCCNV DNA-A and DNA-B wild-type infected clones at 14 dpi. The negative control group displayed no viral disease symptoms (Figure 8A). To analyze the level of SLCCNV accumulation, Southern blotting results showed that the accumulation of SLCCNV-AC5 null mutant was slightly reduced in the upper leaves, as compared with infection with SLCCNV wild-type (Figure 8B). Furthermore, detection of viral DNA accumulation in the SLCCNV-AC5 null mutant DNA-A+ DNA-B-infected plants and the SLCCNV wild-type infected melon plants using quantitative PCR (qPCR) revealed significant differences in the accumulation of DNA-A *AV1* and DNA-B *BV1* genes between the plants inoculated with SLCCNV-AC5 null mutant DNA-A + DNA-B and SLCCNV DNA-A and DNA-B wild-type infectious clones, as determined by Student’s *t*-test (^**^p ≤ 0.01; Figure 8C). Therefore, the AC5 protein was confirmed to be a virulence determinant in melon plants during SLCCNV infection.

## Discussion

Several reports have shown that the DNA-A component of some begomoviruses encodes an AC5 protein, which is a pathogenicity determinant and an RNA silencing suppressor during viral infection (Li et al., 2015, 2021). Here, SLCCNV caused typical *Begomovirus*-induced disease symptoms in melon plants, and its AC5 protein was 172 aa residues long. The length of the AC5 protein varies greatly among begomoviruses. Alignment of the AC5 aa sequences showed that the SLCCNV AC5 protein was 48.11, 45.95, 28.8, 27.06, 11.5, and 9.89% identical to the AC5 proteins encoded by Tomato leaf curl Rajasthan virus - [India: Rajasthan] (ABC70693), Tomato leaf curl Karnataka virus (AHA82240), ToCMoV (AAM12383), WmCSV-[IR] (CAB65502), ALCScV (MG917698), and Mungbean yellow mosaic India virus (CAQ34824), respectively. Furthermore, evolutionary analysis indicated that the SLCCNV AC5 protein was distant from other AC5 proteins. Here, we explored the role of SLCCNV AC5 protein during SLCCNV infection.

Plants have evolved an innate immune response to invading viruses, such as RNA silencing or RNA interference (RNAi; Soosaar et al., 2005) to counteract the host plants’ antiviral defense system. Most viruses encode RSSs that block host RNA silencing *via* RNA silencing pathways (Ding and Voinnet, 2007). Many small RNAs are involved in the defense against *Geminivirus* infection in plants. To overcome these defenses, Geminiviruses have encoded silencing suppressors, such as Croton yellow vein mosaic virus (CYVMV)-encoded RNA silencing suppressors V2, C2, and C4 and beta-satellite-encoded C1 protein encoded by the cognate beta-satellite (Zhai et al., 2022). The Cotton leaf curl multan virus (CLCuMuV) C4 protein suppresses both transcriptional and post-transcriptional gene silencing (Asigul et al., 2018). The ALCScV C5 protein can also suppress PTGS in plants (Li et al., 2021). Here, we not only confirmed that the AC5 protein is a PTGS suppressor, but also analyzed its RNA suppression mechanism. Our results suggest that SLCCNV AC5 interferes with step(s) upstream of the plant RdRP-mediated dsRNA synthesis through the S-PTGS pathway. Furthermore, the active site of the local silencing suppressor of AC5 included the complete C-terminal conserved functional domain Gemini AC5-2; therefore, the C-terminal domain is necessary for the RSS activity of AC5. However, PTGS suppression of MYMIV AC5 was dependent on the AC5 N-terminal domain of the PVX vector (Li et al., 2015). Sequence analysis showed that the lengths of MYMIV AC5 and SLCCNV AC5 were different. Furthermore, MYMIV AC5 included the Gemini AC5-1 domain, and SLCCNV AC5 included the N-terminal Gemini AC5-1 and C-terminal Gemini AC5-2 domains (Li et al., 2015). We speculated that this structural difference might lead to differences in the silencing suppressor activity of these various AC5 proteins.

Some RSSs are necessary for disease symptom formation in plants during virus infection, such as the βC1 protein encoded by the Tomato yellow leaf curl China virus (TYLCCNB; Zhong et al., 2017) and NSs protein encoded by the Tomato spotted wilt virus (Hernan et al., 2018). Notably, AC5 is a symptom determinant of begomoviruses. There are two ideas underlying this phenomenon. One view is that AC5 is not important for viral infection (Kheyr-Pour et al., 2000). During Tomato chlorotic mottle virus (ToCMoV)-[MG-Bt]-DNA-A-mediated infection, AC5 is a PTGS suppressor that is not essential for virus infection (Fontenellea et al., 2007). AC5 mutation analysis of two Tomato leaf deformation virus (ToLDeV) isolates appeared to play no role in viral infectivity and symptom development, whereas an AC5-null mutant of the PA10-3 isolate induced less severe symptoms in *Agrobacterium*-infiltrated *N. benthamiana* leaves (Melgarejo et al., 2013). In contrast, AC5 plays an important role in MYMIV infection (Li et al., 2015). Here, we showed that AC5 enhanced the mRNA accumulation of PVX *cp* in *N. benthamiana* plants and was involved in symptom severity, which was consistent with the previously reported pathogenic roles of their orthologs from other begomoviruses. For example, ALCScV C5 is a virulence factor that contributes to viral infection (Li et al., 2021). The C2 protein of Bhendi yellow vein mosaic virus plays an important role in symptom determination and viral DNA replication in *N. benthamiana* plants (Chandran et al., 2014). In addition, PVX mRNA accumulation of SLCCNV AC5 expressed by pGR106 was higher than that of ALCScV C5 (Li et al., 2021). Moreover, SLCCNV AC5 elicited cell death in *N*. *benthamiana* plants, suggesting that it is involved in the host defense response. Therefore, AC5 is a multifunctional protein involved in SLCCNV infection. This result lays the foundation for further studies on the other functions of AC5 in SLCCNV infection.

In general, the physiological functions of a protein are closely related to its cellular localization. For example, Mulberry mosaic dwarf-associated virus (MMDaV) V2 is localized to both subnuclear foci and the cytoplasm. The V2 protein can inhibit local RNA silencing and the long-distance movement of the RNA silencing signal, but not a short-range spread of the GFP silencing signal in GFP-transgenic *N. benthamiana* 16c plants (Yang et al., 2018). Pepper vein yellows virus (PeVYV) P0 proteins impair local gene silencing and are localized mainly in the nucleus (Wang et al., 2021). The ALCScV C5 protein, a protein that performs multiple functions during viral infection, is localized in both the cytoplasm and nucleus (Li et al., 2021). Similarly, the P6 RNA silencing suppression activity of the Strawberry vein banding virus (SVBV) is completely abolished when its nuclear localization signal is deleted *via* mutagenesis (Feng et al., 2018). Here, the subcellular localization of AC5 in SLCCNV was detected, and the role of the NLS of the AC5 genes was also determined. Therefore, nucleocytoplasmic shuttling of SLCCNV AC5 may regulate viral replication in host plants. In conclusion, the multiple functions of the AC5 gene of SLCCNV involve symptom attenuation and reduced viral replication during SLCCNV infection in host plants. For future research, the identification of host factors interacting with the AC5 protein, such as the nuclear shuttle protein (Martins et al., 2020), would deepen our understanding of SLCCNV virulence mechanisms and provide new clues for the development of disease management strategies in plants.

## Data availability statement

The original contributions presented in the study are included in the article/Supplementary material; further inquiries can be directed to the corresponding authors.

## Author contributions

HW, NH, and QG conceived and designed the study and wrote the paper. HW performed the main experiments. ML, BP, BK, and LL performed some experiments and involved in data analyses. All authors contributed to the article and approved the submitted version.

## Funding

The work was supported by grants from China Agriculture Research System of MOF and MARA(CARS-25), the Agricultural Science and Technology Innovation Program (CAAS-ASTIP-2022-ZFRI-09), the Central Public-Interest Scientific Institution Basal Research Fund (No. 1610192022105), and National Natural Science Foundation of China (No. U21A20229 and 31701941).

## Conflict of interest

The authors declare that the research was conducted in the absence of any commercial or financial relationships that could be construed as a potential conflict of interest.

## Publisher’s note

All claims expressed in this article are solely those of the authors and do not necessarily represent those of their affiliated organizations, or those of the publisher, the editors and the reviewers. Any product that may be evaluated in this article, or claim that may be made by its manufacturer, is not guaranteed or endorsed by the publisher.

## Footnotes

^1^ https://www.ncbi.nlm.nih.gov/Structure/cdd/wrpsb.cgi

^2^ http://nls-mapper.iab.keio.ac.jp/cgi-bin/NLS_Mapper_form.cgi

## References

[ref1] Asigul I. Yakupjan H. Wang Y. Li H. Qian L. Han T.. (2018). Cotton leaf curl Multan virus C4 protein suppresses both transcriptional and post-transcriptional gene silencing by interacting with sam synthetase. PLoS Pathog. 14:e1007282. doi: 10.1371/journal.ppat.1007282 30157283 PMC6133388

[ref2] Chandran S. A. Jeyabharathy C. Usha R. (2014). The C2 protein of bhendi yellow vein mosaic virus plays an important role in symptom determination and virus replication. Virus Genes 48, 203–207. doi: 10.1007/s11262-013-0992-1, PMID: 24122068

[ref3] Ding S. W. Voinnet O. (2007). Antiviral immunity directed by small RNAs. Cell 130, 413–426. doi: 10.1016/j.cell.2007.07.039, PMID: 17693253 PMC2703654

[ref4] Feng M. Zuo D. Jiang X. Shuai L. Jing C. Jiang L.. (2018). Identification of strawberry vein banding virus encoded p6 as an RNA silencing suppressor. Virology 520, 103–110. doi: 10.1016/j.virol.2018.05.003, PMID: 29843054

[ref5] Fondong V. N. (2013). Geminivirus protein structure and function. Mol. Plant Pathol. 14, 635–649. doi: 10.1111/mpp.12032, PMID: 23615043 PMC6638828

[ref6] Fontenellea M. R. Luz D. F. Gomes A. P. S. Florentino L. H. Zerbini F. M. Fontes E. P. B. (2007). Functional analysis of the naturally recombinant DNA-A of the bipartite begomovirus tomato chlorotic mottle virus. Virus Res. 126, 262–267. doi: 10.1016/j.virusres.2007.02.009, PMID: 17367887

[ref7] Hanley-Bowdoin L. Bejarano E. R. Robertson D. Mansoor S. (2013). Geminiviruses: masters at redirecting and reprogramming plant processes. Nat. Rev. Microbiol. 11, 777–788. doi: 10.1038/nrmicro3117, PMID: 24100361

[ref8] Hernan G. R. Sergio G. P. Patricia H. M. (2018). Tomato spotted wilt virus nss protein supports infection and systemic movement of a potyvirus and is a symptom determinant. Viruses 10, 129. doi: 10.3390/v10030129, PMID: 29538326 PMC5869522

[ref9] Kheyr-Pour A. Bananej K. Dafalla G. A. Caciagli P. Gronenborn B. (2000). Watermelon chlorotic stunt virus from the Sudan and Iran: sequence comparisons and identification of a whitefly-transmission determinant. Phytopathology 90, 629–635. doi: 10.1094/PHYTO.2000.90.6.629, PMID: 18944543

[ref10] Lakatos L. Szittya G. Silhavy D. Burgyan J. (2004). Molecular mechanism of RNA silencing suppression mediated by p19 protein of tombusviruses. EMBO J. 23, 876–884. doi: 10.1038/sj.emboj.7600096, PMID: 14976549 PMC381004

[ref11] Li P. Su F. Meng Q. Yu H. Ling Q. (2021). The C5 protein encoded by ageratum leaf curl Sichuan virus is a virulence factor and contributes to the virus infection. Mol. Plant Pathol. 22, 1149–1158. doi: 10.1111/mpp.13103, PMID: 34219358 PMC8359000

[ref12] Li F. Xu X. Huang C. Gu Z. Cao L. Hu T.. (2015). The AC5 protein encoded by mungbean yellow mosaic India virus is a pathogenicity determinant that suppresses RNA silencing-based antiviral defenses. New Phytol. 208, 555–569. doi: 10.1111/nph.13473, PMID: 26010321

[ref13] Lu Q. Y. Yang L. Huang J. Zheng L. Sun X. (2018). Identification and subcellular location of an RNA silencing suppressor encoded by mulberry crinkle leaf virus. Virology 526, 45–51. doi: 10.1016/j.virol.2018.10.007 30342301

[ref14] Maina S. Edwards O. R. Almeida L. D. Ximenes A. Jones R. (2017). First complete squash leaf curl China virus genomic segment DNA-A sequence from East Timor. Genome Announc. 5, e00483–e00417. doi: 10.1128/genomeA.00483-17, PMID: 28619789 PMC5473258

[ref15] Martins L. G. C. Raimundo G. A. S. Ribeiro N. G. A. Silva J. C. F. Euclydes N. C. Loriato V. A. P.. (2020). A *begomovirus* nuclear shuttle protein-interacting immune hub: hijacking host transport activities and suppressing incompatible functions. Front. Plant Sci. 11:398. doi: 10.3389/fpls.2020.00398, PMID: 32322262 PMC7156597

[ref16] Melgarejo T. A. Kon T. Rojas M. R. Paz-Carrasco L. Zerbini F. M. Gilbertson R. L. (2013). Characterization of a new world monopartite begomovirus causing leaf curl disease of tomato in Ecuador and Peru reveals a new direction in geminivirus evolution. J. Virol. 87, 5397–5413. doi: 10.1128/JVI.00234-13, PMID: 23468482 PMC3648196

[ref17] Ocampo T. O. Peralta S. M. G. Bacheller N. Uiterwaal S. Garcia-Ruiz H. (2015). Antiviral rna silencing suppression activity of tomato spotted wilt virus NSs protein. Genet. Mol. Res. 15:8625. doi: 10.4238/gmr.15028625, PMID: 27323202 PMC6097843

[ref18] Riyaz S. U. M. Deepan S. Dharanivasan G. Jesse M. I. Muthuramalingam R. Kathiravan K. (2013). First report on a variant of squash leaf curl China virus (SLCCNV) infecting Benincasa hispida in India. New Dis. Rep. 28, 20. doi: 10.5197/j.2044-0588.2013.028.020

[ref19] Ruiz M. T. Voinnet O. Baulcombe D. C. (1998). Initiation and maintenance of virus-induced gene silencing. Plant Cell 10, 937–946. doi: 10.1105/tpc.10.6.937, PMID: 9634582 PMC144041

[ref20] Saritha R. K. Bag T. K. Loganathan M. Rai A. B. Rai M. (2011). First report of squash leaf curl China virus causing mosaic symptoms on summer squash (*Cucurbita pepo*) grown in Varanasi district of India. Arch. Phytopathol. Plant Protect. 44, 179–185. doi: 10.1080/03235400902952301

[ref21] Sawangjit S. (2009). The complete nucleotide sequence of squash leaf curl China virus- [wax gourd] and its phylogenetic relationship to other *Geminiviruses*. ScienceAsia 35, 131–136. doi: 10.2306/scienceasia1513-1874.2009.35'.131

[ref22] Sharma P. Ikegami M. (2010). Tomato leaf curl java virus v2 protein is a determinant of virulence, hypersensitive response and suppression of posttranscriptional gene silencing. Virology 396, 85–93. doi: 10.1016/j.virol.2009.10.012, PMID: 19896687

[ref23] Siddell S. G. Walker P. J. Lefkowitz E. J. Mushegian A. R. Adams M. J. Dutilh B. E.. (2019). Additional changes to taxonomy ratified in a special vote by the international committee on taxonomy of viruses (october 2018). Arch. Virol. 164, 943–946. doi: 10.1007/s00705-018-04136-2, PMID: 30663020

[ref24] Soosaar J. Burch-Smith T. M. Dinesh-Kumar S. P. (2005). Mechanisms of plant resistance to viruses. Nat. Rev. Microbiol. 3, 789–798. doi: 10.1038/nrmicro1239 16132037

[ref25] Tamura K. Stecher G. Peterson D. Filipski A. Kumar S. (2013). Mega6: molecular evolutionary genetics analysis version 6.0. Mol. Biol. Evol. 30, 2725–2729. doi: 10.1093/molbev/mst197, PMID: 24132122 PMC3840312

[ref26] Wang L. S. Tian P. J. Yang X. L. Zhou X. P. Zhang S. B. Chun L.. (2021). Key amino acids for pepper vein yellows virus P0 protein pathogenicity, gene silencing, and subcellular localization. Front. Microbiol. 12:680658. doi: 10.3389/fmicb.2021.680658, PMID: 34589062 PMC8475269

[ref27] Wu H. J. Li M. Hong N. Peng B. Gu Q. S. (2020). Molecular and biological characterization of melon-infecting squash leaf curl China virus in China. J. Integr. Agric. 19, 570–577. doi: 10.1016/S2095-3119(19)62642-0

[ref28] Xiong R. Y. Wu J. X. Zhou Y. J. Zhou X. P. (2009). Characterization and subcellular localization of an RNA silencing suppressor encoded by rice stripe tenuivirus. Virology 387, 29–40. doi: 10.1016/j.virol.2009.01.045, PMID: 19251298

[ref29] Yang Z. R. Huang Y. Yang J. L. Yao S. Z. Zhao K. Wang D. H.. (2020). Jasmonate signaling enhances RNA silencing and antiviral defense in rice. Cell Host Microbe 28, 89–103.e8. doi: 10.1016/j.chom.2020.05.001, PMID: 32504578

[ref30] Yang X. Ren Y. Sun S. Wang D. Zhang F. Li D.. (2018). Identification of the potential virulence factors and RNA silencing suppressors of mulberry mosaic dwarf-associated geminivirus. Viruses 10, 472. doi: 10.3390/v10090472, PMID: 30177616 PMC6163789

[ref31] Zhai Y. Roy A. Peng H. Mullendore D. L. Kaur G. Mandal B.. (2022). Identification and functional analysis of four RNA silencing suppressors in *Begomovirus* croton yellow vein mosaic virus. Front. Plant Sci. 12:768800. doi: 10.3389/fpls.2021.768800, PMID: 35069624 PMC8777275

[ref32] Zhong X. Wang Z. Q. Xiao R. Cao L. Wang Y. Xie Y.. (2017). Mimic phosphorylation of a βc1 encoded by tylccnb impairs its functions as a viral suppressor of RNA silencing and a symptom determinant. J. Virol. 91, e00300–e00317. doi: 10.1128/JVI.00300-17, PMID: 28539450 PMC5533934

