# Supplementary material for: AC5 protein encoded by squash leaf curl China virus is an RNA silencing suppressor and a virulence determinant
Source: Front Microbiol. 2022 Aug 19;13:980147. doi: 10.3389/fmicb.2022.980147 (PMC9437540; doi:10.3389/fmicb.2022.980147)
Supplement: Supplementary file 1 [file Table_1.DOCX]

Supplementary Table 1 Primers used in the study.

| Names | Sequences of primers (5’ to 3’) |
| --- | --- |
| SLCCNV-AC5 null-mutant-F | ATA CCA AGT GCT GCG CAA ATG GCA cGG CAC TGT GAC GGG AGG AAC AT |
| SLCCNV-AC5 null-mutant-R | ATG TTC CTC CCG TCA CAG TGC CGT gCC ATT TGC GCA GCA CTT GGT A T |
| SLCCNV-A-F | GTC GCG TAT ACA GGG TTC GAT GCG TG |
| SLCCNV-A-R | ATG TCG AAG CGA CCA GCC GAT ATT |
| SLCCNV-B-F-478 | ATG GCT TTT ACC GGA TCA TAT AC |
| SLCCNV-B-R-1284 | ATT AAC CAA TGT AAT TTA GCA TTA C |
| p35S-AC5(1-132)-F | ACTGTATCAATTCGAGCTCGGTACC ATG CCATTTGCGCAGCACTT |
| p35S -AC5(1-132)-R | TAAAACGACGGCCAGTGCCAAGCTTT CAGTTCGAGACCGTCGTCCTAC |
| p35S -AC5(133-287)-F | ACTGTATCAATTCGAGCTCGGTACC ATG CAGAAAAAACATGACACT |
| p35S -AC5(133-287)-R | TAAAACGACGGCCAGTGCCAAGCTTT CAAGGTGATGTGTATTAGTGATG |
| p35S -AC5(288-519)-F | ACTGTATCAATTCGAGCTCGGTACC ATG TACCAATATGGGAGACAT |
| p35S -AC5(288-519)-R | TAAAACGACG GCCAGTGCCAAGCTT TCAACTCCCGCGTCGAAAG |
| pGR106-AC5-F | GTCAGCACCAGCTAGCATCGATATGCCATTTG CGCAGCACTT the sequences of underlined parts were *Cla*I |
| pGR 106-AC5-R | TCAAGCTTATCGGCGGTCGAC TCAACTCCCG CGTCGAAAG the sequences of underlined parts were *Sal*I |
| AC5-probe-F | ATG CCA TTT GCG CAG CAC TTG G |
| T7-AC5-probe-R | TAATACGACTCACTATAGGG TCA ACT CCC G CG TCG AAA GT A, the underlined part is the T7 sequences. |
| GFP-probe-F | AGTAAAG GAGAAGAACT TTTcact |
| T7-GFP-probe-R | TAATACGACTCACTATAGGG TTTGTAT AGTTCATCCA TGCCATG, the underlined part is the T7 sequences. |
| PVX-cp-F | ATGTCAGCACCAGCTAGCA |
| PVX-cp-T7-R | TAATACGACTCACTATAGGGG ATTGTGCCCTGGCCTTTGTA, the underlined part is the T7 sequences. |
| DNA-A-AV1-F1 | TAGTGATGTTACACGAGGAACCG |
| DNA-A-AV1-R1 | AAATCCTGTGGAGTTCCTGTAGG |
| DNA-B-BV1-F1 | GGTGCTCAATCAAACGTCCATAC |
| DNA-B-BV1-R1 | GTATTTGAGTACAAACCGGTGGC |
| melonactin-F | ATGGTCAAGGCTGGATTTGC |
| melonactin-R | TGAGCTTCATCACCAACATAGGC |
| G2 | CTTTCTCATCTTTTCACTTCTCCTATCATTATCCTCGGCC, with a DIG biotin added at the 5´ends |
| G3 | GAATTCAGTAAAGGAGAAGAACTTTTCACTGGAGTTGTC, with a DIG biotin added at the 5´ends |
| F1 | AATTTTCTGTCAGTGGAGAGGGTGAAGGTGATGCAACATA, with a DIG biotin added at the 5´ends |
| F2 | CGGAAAACTTACCCTTAAATTTATTTGCACTACTGGAAAA, with a DIG biotin added at the 5´ends |
| F3 | CTACCTGTTCCATGGCCAACACTTGTCACTACTTTCTCTT, with a DIG biotin added at the 5´ends |
| P1 | CAAGTTGGAATACAACTACAACTCCCACAACGTATACATC, with a DIG biotin added at the 5´ends |
| PVXcp-F | CTCAACTACCACGAAAACTGC |
| PVXcp-R | TGTGCACACCTCTTTGATTGC |
| Nb-actin-F | CTTGAAACAGCAAAGACCAGC |
| Nb-actin-R | CATCCTATCAGCAATGCCCG |
| AC5^Δ102-115 aa^-F | ACAGCCCCTTGGCACGTCGGG |
| AC5^Δ102-115 aa^-R | CCGACGTGCC AAGGGGCTGT GTCTCCCATA TTGGTAAGGT GAT |
